# First record of *Thecturota tenuissima* Casey from Canada (Coleoptera, Staphylinidae, Aleocharinae)

**DOI:** 10.3897/zookeys.702.19963

**Published:** 2017-09-25

**Authors:** Jan Klimaszewski, Tim Struyve, Caroline Bourdon, Julie-Anne Dorval

**Affiliations:** 1 Natural Resources Canada, Canadian Forest Service, Laurentian Forestry Centre, 1055 du P.E.P.S., P.O. Box 10380, Stn. Sainte-Foy, Québec, G1V 4C7, Canada; 2 Leuvensesteenweg 187, 2800 Mechelen, Belgium

**Keywords:** Canada, new record, *Thecturota*, Aleocharinae, Staphylinidae, Coleoptera

## Abstract

*Thecturota tenuissima* Casey, is reported for the first time from Canada, based on records from Ontario and Quebec. It was originally described from Rhode Island, USA, and no other records of this species in North America were published since that time. The specimens from Canada were captured by car netting. We provide here a redescription of this species and never before published images of habitus, tergite, and sternite VIII of both sexes, median lobe of aedeagus and spermatheca. The features distinguishing *T. tenuissima* from *T. capito* Casey (=*pusio* (Casey)), the only other species reported in Canada, are provided and illustrated.

## Introduction

*Thecturota* Casey, is a genus of the tribe Homalotini Heer, with 5 valid species in the Nearctic region (Fenyes 1920). Two species, *T. ruficollis* Casey and *T. subtilior* (Bernhauer), occur in southwestern USA, Arizona, southern California, and Nevada. The remaining three valid species, *T. capito* Casey, *T. demissa* Casey, *T. tenuissima* Casey, are reported from central, eastern, and southern USA (Rhode Island, New York, Iowa, Indiana, Virginia, and Texas), and eastern Canada. We have examined the type specimens of the three eastern species. The objective of this paper is to publish a new Canadian record of *T. tenuissima* with redescription and images of body and genital structures and to provide a key for identification of the Canadian species. It is important to note that the shape of the spermatheca is the best criterion for separating species of this genus.

## Materials and methods

All specimens in this study were dissected to examine the genital structures. Extracted genital structures were dehydrated in absolute alcohol, mounted in Canada balsam on celluloid micro-slides, and pinned with the specimen from which they originated. Images of the entire body and the genital structures were taken using an image processing system (Nikon SMZ 1500 stereoscopic microscope; Nikon Digital Camera DXM 1200F, and Adobe Photoshop software).

Morphological terminology mainly follows that used by Seevers (1978). The ventral side of the median lobe of the aedeagus is considered to be the side of the bulbus containing the foramen mediale, the entrance of the ductus ejaculatorius, and the adjacent ventral side of the tubus of the median lobe with the internal sac and its structures (this part is referred to as the parameral side in some recent publications); the opposite side is referred to as the dorsal part.

### Depository/institutional abbreviations

**LFC** Natural Resources Canada, Canadian Forest Service, Laurentian Forestry Centre, R. Martineau Insectarium, Quebec City, Quebec, Canada.

**TSC** Tim Struyve collection, Leuvensesteenweg. 187, 2800 Mechelen, Belgium.

**USNM** United States National Museum, Washington, D.C, USA.

## Systematic treatments

### Genus Thecturota

Taxon classificationAnimaliaColeopteraStaphylinidae

Casey, 1893

Figs 1–8 , 9–15

Casey 1893, 1911, Seevers 1978, Ashe 2001

#### Diagnosis.

Body slender, narrow, linear, small, length 1.0–1.7 mm; pubescence on pronotum and elytra directed laterad; head subquadrate with angular posterior tempora, as long and at least as wide as pronotum; eyes shorter than length of temples; antennomeres V-X strongly transverse and slightly incrassate apically, pronotum small, subquadrate or 1.2 times as wide as long, broadest subapically, pubescence directed anteriad and laterad from midline of disc; elytra short, at suture about as long as pronotum or slightly longer; abdomen subparallel. Male tergite VIII truncate apically; tubus of median lobe of aedeagus simple in form, its venter arcuate; internal sac structures inconspicuous; spermatheca small, capsule subspherical with small or without apical invagination, stem very short.

#### Key to Canadian species of *Thecturota*

**Table:** 

1	Male tergite VIII nearly two times as wide as long (Fig. 4); male sternite VIII about one fourth wider than long (Fig. 5); spermatheca with capsule pitcher-shaped with narrow apical invagination (Fig. 8)	***Thecturota capito* Casey**
–	Male tergite VIII nearly as wide as long (Fig. 11); male sternite VIII about as wide as long (Fig. 12; spermatheca with capsule subspherical lacking apical invagination (Fig. 15)	***Thecturota tenuissima* Casey**

### 1. Thecturota capito

Taxon classificationAnimaliaColeopteraStaphylinidae

Casey

Figs 1–8

Thecturota capito Casey, 1893: 358. Casey 1911, Fenyes 1920, Moore and Legner 1975, Seevers 1978, Brunke et al. 2012 (as T. pusio (Casey)).
Oligurota pusio Casey, 1893: 362. Brunke et al. 2012. Synonymized by Fenyes 1920.
Thecturota exigua Casey, 1894: 360. Synonymized by Fenyes 1920.
Thecturota histrio Casey, 1911: 210. Synonymized by Fenyes 1920.
Thecturota laticeps Casey, 1911: 208. Synonymized by Fenyes 1920.

#### Diagnosis.

Body length 1.2–1.6 mm; body narrowly subparallel, linear, color variable: reddish brown with head and abdomen dark brown to piceous, or body piceous with reddish brown elytra, and yellowish-red legs, basal antennomeres and tip of abdomen; integument finely punctate; head subquadrate, larger than pronotum, with postocular area longer than diameter of eye, hind angles angular and rounded; antennomeres IV-X transverse and VI-X strongly transverse and about 3 times wider than long; pronotum broadest in apical third, narrow at base, pubescence directed laterad from midline of disc; elytra subparallel, at suture about as long as pronotum; abdomen subparallel, slightly broadening posterad. MALE: tergite VIII nearly two times as wide as long (Fig. 4); sternite VIII about one fourth wider than long (Fig. 5); median lobe of aedeagus with narrowly oval bulbus and subparallel tubus in dorsal view (Fig. 3); in lateral view tubus broadly arcuate ventrally, with apex narrow and rounded (Fig. 2). FEMALE: tergite VIII nearly two times as wide as long (Fig. 6); sternite VIII rounded apically (Fig. 7); spermatheca with large pitcher-shaped capsule and narrow apical invagination, stem very short (Fig. 8).

**Figures 1–8.**
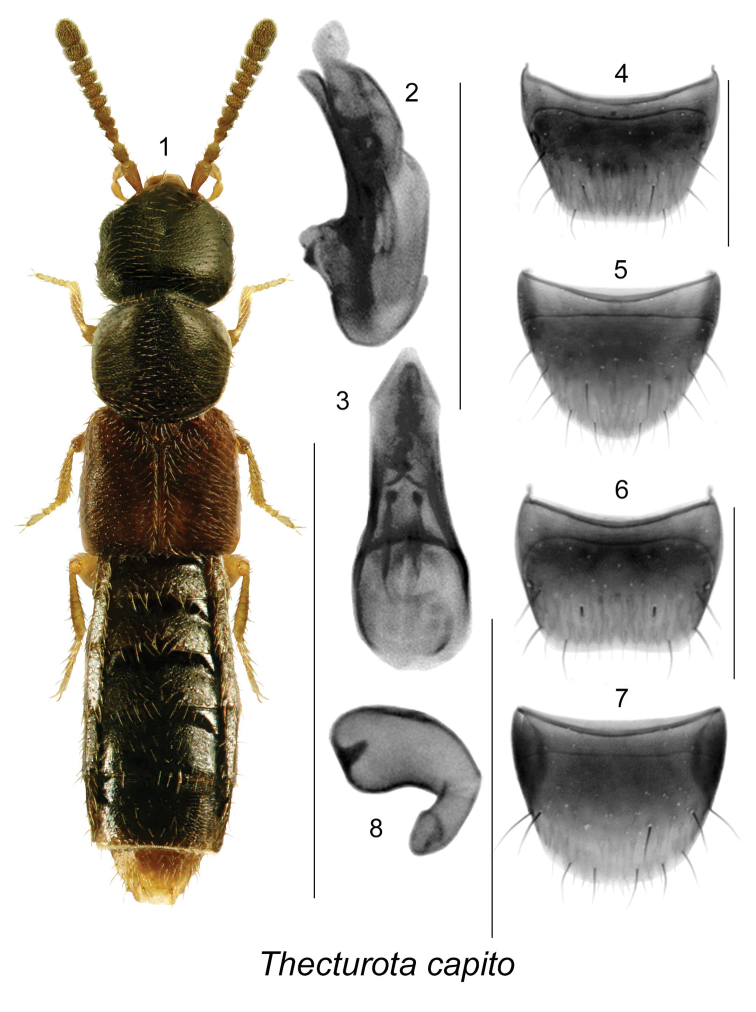
*Thecturota capito* Casey: **1** habitus in dorsal view **2** median lobe of aedeagus in lateral view **3** median lobe of aedeagus in dorsal view **4** male tergite VIII **5** male sternite VIII **6** female tergite VIII **7** female sternite VIII **8** spermatheca. Scale bars: 1 mm for habitus; 0.2 mm for remaining structures.

#### Distribution.

**Origin**: Nearctic. **Canada**: **ON**. **USA**: IA, IN, TX, VA.

#### Collection and habitat data.

**Habitat**: oak savanna from leaf, log, and grass litter. **Collecting period**: X. **Collecting method**: Berlese extraction of leaf, log, and grass litter. Live adults of this species were extremely slow-moving and the use of a Berlese funnel likely facilitated the capture of this minute beetle.

#### Comments.

*Thecturota capito* Casey was recorded from Canada (ON) for the first time under a synonymic name *T. pusio* (Casey) by Brunke et al. (2012). Fenyes (1920) and Moore and Legner (1975) listed *T. pusio*, originally named as *Oligurota pusio* Casey 1893: 362, as a synonym of *T. capito* Casey 1893: 358. We overlooked this fact in Brunke et al. 2012, and now it is corrected. In the original description, Casey also mentioned specimens from AZ (Tucson).

### 2. Thecturota tenuissima

Taxon classificationAnimaliaColeopteraStaphylinidae

Casey

Figs 9–15

Thecturota tenuissima Casey, 1893: 358. Fenyes 1918, 1920, Moore and Legner 1975, Seevers 1978, Sikes 2004.

#### Diagnosis.

Body length 1.5–1.7 mm; body narrowly subparallel, linear, yellowish brown with head, most of antennae, and apical part of abdomen dark brown to piceous, or body light brown with darker head and posterior abdomen or pronotum and elytra brown and remainder of the body dark brown to almost black; integument finely punctate; head subquadrate, larger than pronotum, with postocular area longer than diameter of eye, hind angles angular and rounded; antennomeres IV-X transverse and VI-X strongly transverse and about 2 times wider than long; pronotum broadest in apical third, narrow at base, pubescence directed laterad from midline of disc; elytra subparallel, at suture slightly longer than pronotum; abdomen subparallel, slightly broadening posterad. MALE: tergite VIII nearly as wide as long (Fig. 11); sternite VIII about as wide as long (Fig. 12); median lobe of aedeagus with tubus arcuate ventrally and narrow at apex in lateral view (Fig. 10). FEMALE: tergite VIII subquadrate (Fig. 13); sternite VIII rounded apically and in some specimens slightly emarginate medially (Fig. 14); spermatheca with subspherical capsule and without apical invagination, stem very short and sinuate (Fig. 15).

**Figures 9–16.**
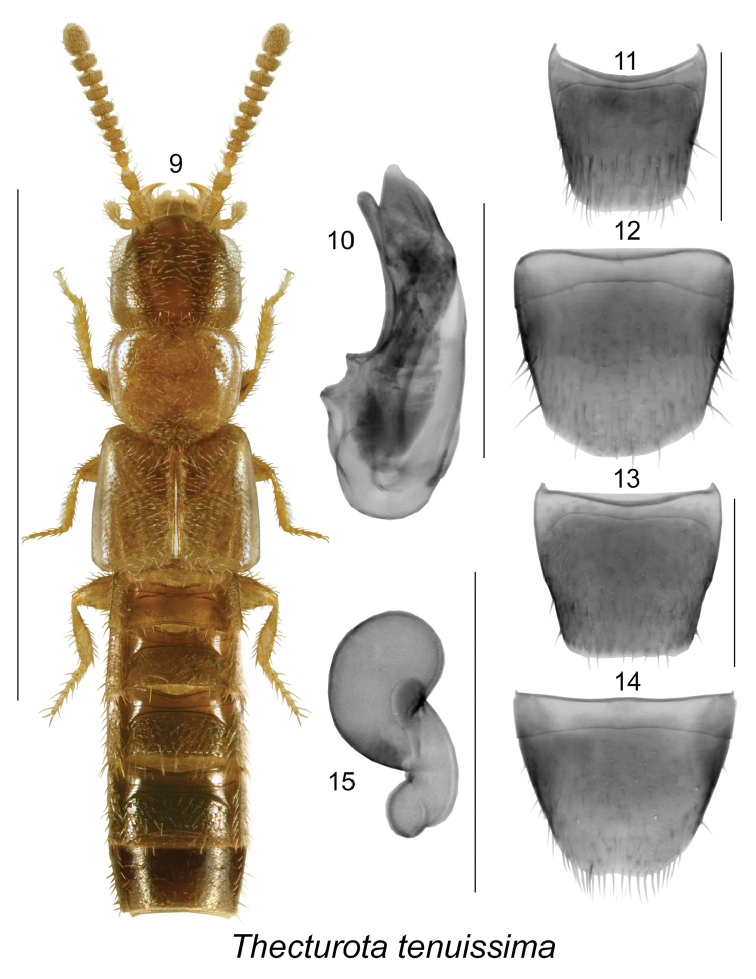
*Thecturota tenuissima* Casey: **9** habitus in dorsal view **10** median lobe of aedeagus in lateral and dorsal view **11** male tergite VIII **12** male sternite VIII **13** female tergite VIII **14** female sternite VIII **15** spermatheca. Scale bars: 1 mm for habitus; 0.2 mm for remaining structures.

#### Distribution.

**Origin**: Nearctic. **Canada**: **ON**, **QC**. **USA**: RI.

#### Collection and habitat data.

**Habitat**: unspecified forests. **Collecting period**: VII. **Collecting method**: car netting.

#### New locality data.

CANADA, **Quebec**: Oka, ~ 45.49°N, 74.01°W, 12.VII.2016, car netting, Tim Struyve (LFC, TSC) 1 male, 15 females; Saint-Joseph-du-Lac, ~ 45.53°N, 74.02°W, 11.VII.2016, car netting, Tim Struyve (LFC, TSC) 4 males, 1 female; Port Rowan, ~ 42.62°N, 80.53°W, car netting, Tim Struyve (LFC, TSC) 2 females. **Ontario**: Algonquin Provincial Park near Petawawa, ~ 45.87°N, 77.33°W, car netting, Tim Struyve (LFC, TSC) 1 male, 1 female.

## Supplementary Material

XML Treatment for Thecturota

XML Treatment for Thecturota capito

XML Treatment for Thecturota tenuissima
